# Epidemiological features and risk factors of human psittacosis in Hangzhou City, eastern China

**DOI:** 10.3389/fpubh.2025.1512841

**Published:** 2025-02-06

**Authors:** Zhou Sun, Ke Xu, Liangliang Huo, Xingliang Zhang, Yi Wang, Yonghui Gong, Bingbing Chen

**Affiliations:** ^1^Hangzhou Center for Disease Control and Prevention (Hangzhou Health Supervision Institution), Hangzhou, China; ^2^The Affiliated Hospital of Hangzhou Normal University, Hangzhou, China

**Keywords:** epidemiological, human psittacosis, *Chlamydia psittaci*, transmission, risk factors

## Abstract

**Objectives:**

This study aimed to investigate the epidemiological characteristics and risk factors associated with human psittacosis in Hangzhou city, eastern China.

**Methods:**

The human psittacosis data from 2021 to 2024 were obtained from the China information system for diseases control and prevention infectious disease surveillance system. Epidemiological investigations were carried out on the patients' past medical history, clinical manifestations, chest CT results and treatment status. A community-based 1:3 matched case-control study was performed to investigate the risk factors associated with *Chlamydia psittaci* infection.

**Results:**

During the study period, 137 confirmed cases of human psittacosis were identified through laboratory tests, of which 24 (17.52%) were classified as critical cases, including one fatality. The epidemic curve indicated that the majority of cases occurred between October and March. Among the cases, 48.91% were female, and the median age was 63 years. There were more female cases among those aged <60 years, while there were more male cases among those aged ≥60 years. Multivariate logistic regression analysis revealed that the presence of bird habitats within 500 m of the living area [odds ratio (OR) = 3.81, 95% confidence interval (CI) = 2.19–6.61], parrots kept (OR = 2.95, 95%CI = 1.10–7.89) and poultry kept (OR = 2.15, 95%CI = 1.02–4.53) remained significantly associated with the risk of disease infection.

**Conclusions:**

Human psittacosis has become a notable public health concern in Hangzhou city, with an increase in psittacosis cases reported in recent years. Exposure to poultry, birds, or environments contaminated with *Chlamydia psittaci* was associated with infection. Urgent actions to reduce psittacosis cases and mitigate the impact of outbreaks are needed, including strengthening surveillance, raising public awareness, and promoting collaboration between the agricultural and health sectors.

## 1 Introduction

*Chlamydia psittacosis* (*C. psittaci*) is a zoonotic pathogen that affects humans, birds and various animal populations (1). *C. psittaci* is currently classified into 15 different outer membrane protein A (ompA) genotypes: A to F, E/B, WC, M56, 1V, 6N, Mat116, R54, YP54, and CPX0308 (2). Humans are susceptible to infection by any genotype of *C. psittaci*, but certain genotypes, such as genotype A, appear to be more frequently linked to severe illness in infected patients compared to others (3). A variety of clinical manifestations have been documented in humans with psittacosis, ranging from the more common subclinical or brief, self-resolving flu-like symptoms to the rarer but severe cases of fulminant psittacosis, characterized by multi-organ failure. When treated appropriately, the infection is rarely fatal (4, 5). Although direct human-to-human transmission is uncommon, the disease is primarily contracted by inhaling bacteria from bird droppings or secretions through close contact. Individuals who interact with birds as part of their leisure or professional activities, including pet bird enthusiasts and breeders, employees in pet shops, zoo workers, poultry industry workers, veterinarians, and wildlife keepers, were at the highest risk (6).

Although psittacosis is a rare disease, fewer than 10 cases were reported annually in the United States from 2006 to 2012 (7, 8). Sporadic outbreaks of psittacosis have also been documented in certain locations (9). Due to restricted testing and deficiencies in historical diagnostic methods, the reported figures may not accurately reflect the true incidence of human cases. In China, most human psittacosis cases were sporadic. However, a small number of clusters have been reported in recent years (10). Nevertheless, experts believe that there is significant underreporting and potential misdiagnosis of human psittacosis, which may be attributed to insufficient awareness of the disease and restricted testing capabilities.

As metagenomic next-generation sequencing (mNGS) technology has been increasingly utilized in recent years, an increasing number of human psittacosis cases have been identified and reported. Unlike conventional diagnostic methods, mNGS is less affected by antibiotic use and is able to detect rare, novel, and unforeseen pathogens without preconceived biases (11).

Hangzhou, a central city in the Yangtze River Delta region, is situated in eastern China, surrounded by mountains and lush greenery. Although human psittacosis is not considered a notifiable infectious disease in China, cases have been reported in Hangzhou since 2021. The case data were collected and epidemiological investigations were conducted. In this study, we undertook a comprehensive analysis to understand the epidemiological features and risk factors associated with human psittacosis in Hangzhou from 2021 to 2024.

## 2 Materials and methods

### 2.1 Data collection

Data were collected on laboratory-verified cases of human psittacosis from the China information system for diseases control and prevention infectious disease surveillance system from January 1, 2021, to June 30, 2024.

### 2.2 Study design, case, and control definition

The study was a community-based 1:3 matched case-control investigation involving 80 cases and 239 controls. The criteria for the diagnosis of human psittacosis case were as follows: (1) Individuals who meet the diagnostic criteria for community-acquired pneumonia (CAP) according to the Chinese Guidelines for the Diagnosis and Treatment of Community-acquired Pneumonia in Adults (12); (2) The presence of specific gene fragments of *C. psittaci* in samples detected by real-time reverse transcription polymerase chain reaction (RT-PCR) or metagenomic next-generation sequencing (mNGS). The controls were neighbors who had lived in the same community or village as the cases for over 6 months and were no more than 5 years older than the cases. The controls had no respiratory symptoms, such as fever, cough, or chest tightness, in the previous 3 months.

### 2.3 Methodology and content of the survey

The “Questionnaire for human psittacosis case” was independently developed and administered by trained professionals acting as investigators. Face-to-face interviews were conducted with the cases and their families, and their responses were accurately recorded. The control survey was conducted simultaneously by the same researchers. The investigation encompassed general information, details regarding medical consultations and treatments, clinical manifestations, laboratory test results, medical history, family circumstances, and exposure to live birds or poultry.

### 2.4 Sample collection and testing

The samples of alveolar lavage fluid, sputum, and peripheral blood from the patient were collected and transported in a refrigerated container (4–8°C) to the laboratory for testing. Real-time reverse transcription polymerase chain reaction (RT-PCR) and metagenomic next-generation sequencing (mNGS) were employed to detect specific gene fragments of *C. psittaci* in the samples.

### 2.5 Statistical analysis

Data entry was conducted using EpiData 3.1, and database consistency checks were performed. Data cleaning and organization were carried out using WPS Office software (Kingsoft Corporation Limited). The Chi-square test was employed as a univariable regression analysis. Multivariable logistic regression was performed using SPSS version 25.0 (Statistical Product and Service Solutions, Chicago, IL). The interquartile range (IQR), odds ratios (OR), and 95% confidence intervals (CI) were calculated.

## 3 Results

### 3.1 Demographic characteristics of the study population

**Figure 1:**
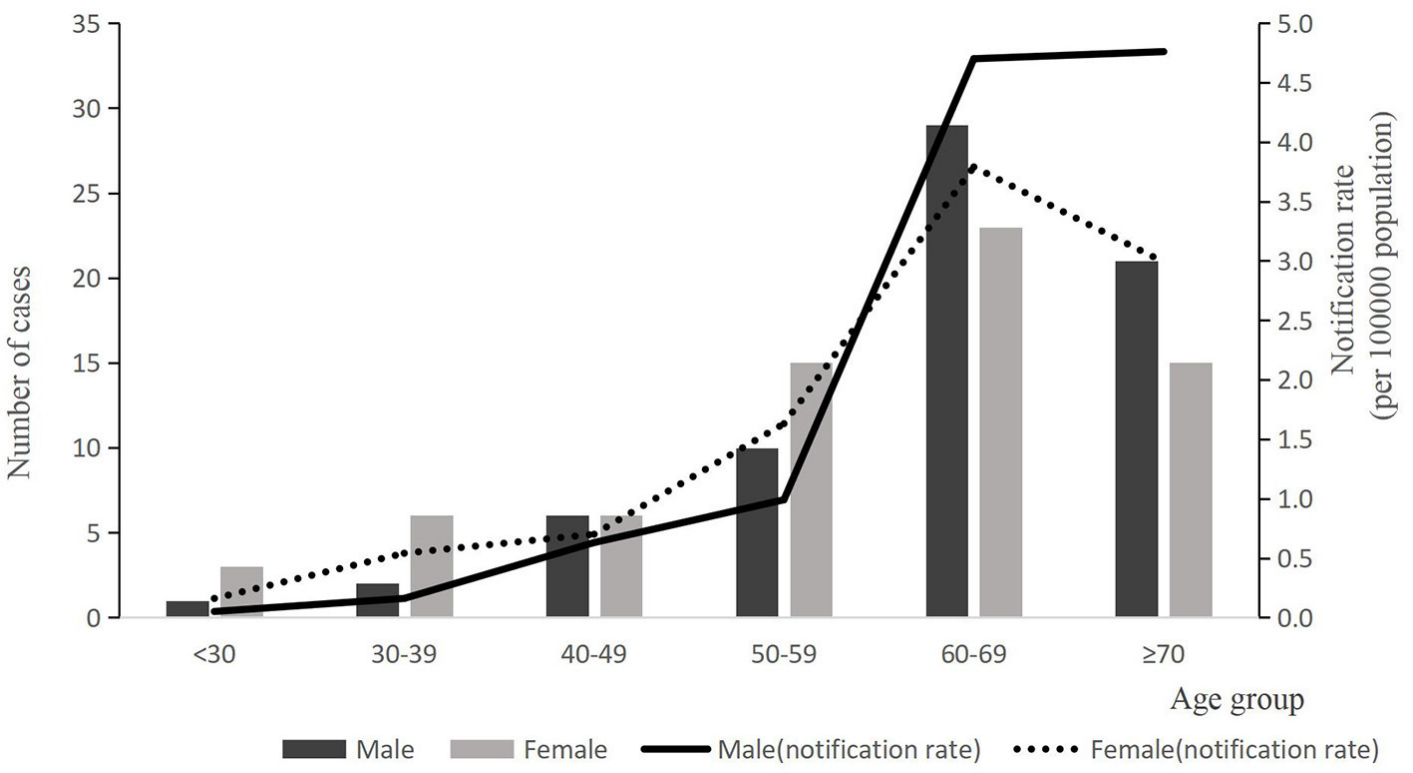
The number of reported case and notification rate of psittacosis by age group of gender, 2021–2024(*n* = 137). Bars express number of cases by age group. Black bar indicates male cases and gray shows female cases. Notification rate per 100,000 population is indicated by lines (solid line indicates male and dotted line indicates female).

A total of 137 laboratory-confirmed cases of human psittacosis were reported during the study period, of which 24 (17.52%) were classified as critical cases. 136 patients improved clinically and were discharged home; however, one patient died. Among the cases, 135 (98.54%) were diagnosed using mNGS, while 2 cases (1.46%) were diagnosed by RT-PCR. Female-confirmed psittacosis cases numbered 67 (48.91%), resulting in a male-to-female ratio of 1.04:1. Notification rates for those aged < 60 years were higher among females, while rates for those aged ≥60 years were higher in males. The median age was 63 years [interquartile range (IQR) = 53–70 years). Eighty eight cases (64.23%) were mainly in the age group ≥60 years and the notification rate was 4.06 per 100,000, which was higher than those aged < 60 years (*P* < 0.05). Most confirmed cases were homemakers, accounting for 46.72% (64 of 137), followed by farmers at 24.82% (34 of 137). Some cases also had a history of chronic medical conditions, including cardiovascular disease (15.33%, 21/137), diabetes (10.22%, 14/137) and others (2.92%, 4/137), as shown in Table 1 and Figure 1.

**Table 1:** Summary of epidemiological characteristics of psittacosis in Hangzhou City from 2021 to 2024.

**Characteristics**	**Total cases (*n =* 137), no. (%)**	**Deaths (*n =* 1)**
Female	67(48.91)	1
**Age**		
Age < 30	4(2.92)	0
30–39	8(5.84)	0
40–49	12(8.76)	0
50–59	25(18.25)	0
60–69	52(37.96)	0
≥70	36(26.28)	1
**Occupation**		
Farmer	34(24.82)	0
Homemaker	64(46.72)	1
Others	39(28.47)	0
**Chronic disease**		
Cardiovascular disease	21(15.33)	1
Diabetes	14(10.22)	0
Others (tumor, chronic bronchitis, liver disease, etc.)	4(2.92)	0
**Temporal distribution (years)**		
2021	9	0
2022	26	1
2023	62	0
2024.1–6	40	0

### 3.2 Temporal trends

**Figure 2:**
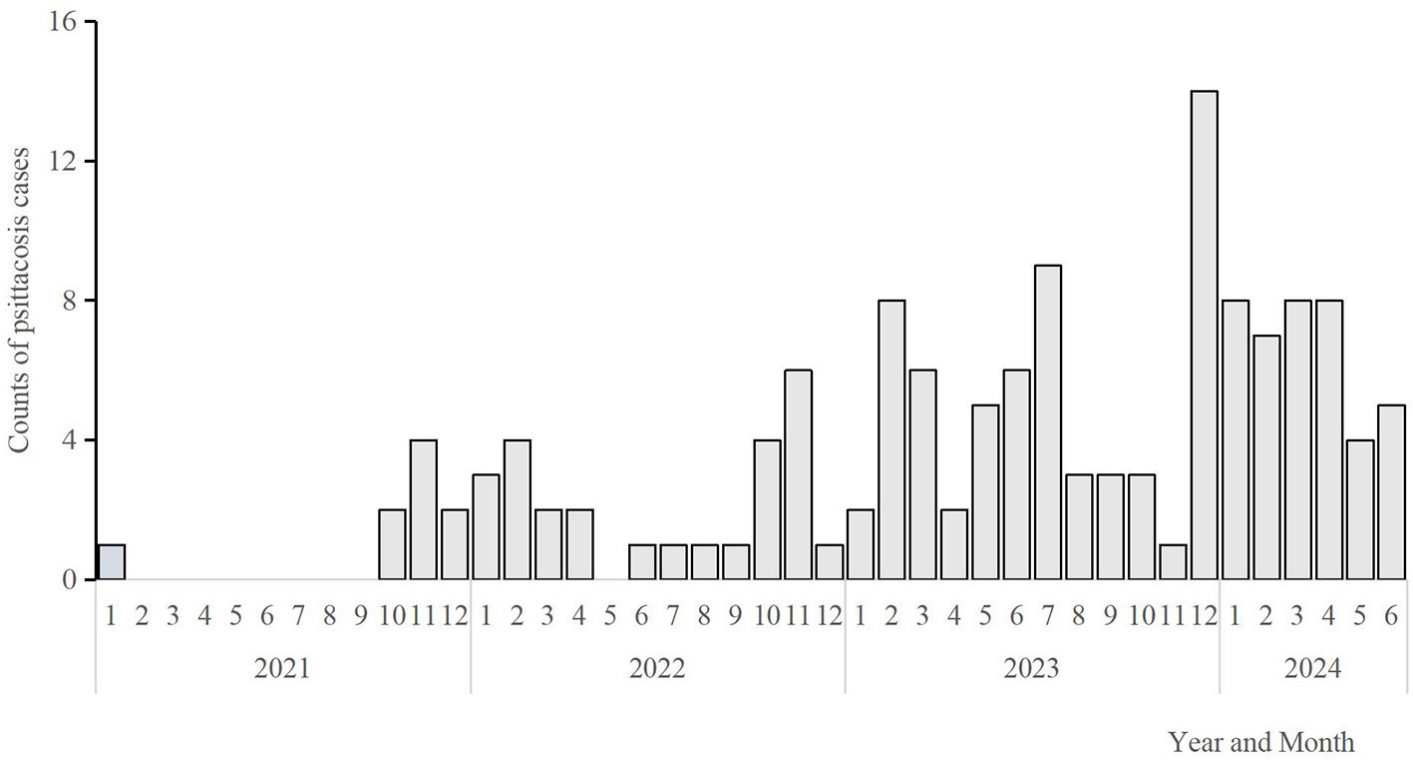
Monthly distribution of Pisstoci cases in Hangzhou City, 2021–2024(*n* = 137). The bars express the number of cases on a monthly basis from January 2021 to June 2024.

The number of confirmed human psittacosis cases from January 1, 2021, to June 30, 2024, was as follows: 9, 26, 62, and 40 cases per year. Confirmed cases were reported monthly. A rapid increase in the number of cases was observed in January, followed by a slowdown in May, as shown in Figure 2. One outbreak was identified in June 2022, involving two female cases in one family that kept two parrots at home.

### 3.3 Symptoms and treatment

The most common clinical symptoms included fever (100%, 137/137), cough (56.20%, 77/137), expectoration (40.15%, 55/137), chest tightness (27.74%, 38/137), fatigue (59.12%, 81/137), limb fatigue (27.74%, 38/137), and headache (23.36%, 32/137). Twenty-four critical patients required endotracheal intubation and mechanical ventilation. Most cases exhibited flake or strip-shaped high-density shadows, and some patients had pleural effusion. We studied 83 cases with results from computed tomography (CT) of the lungs. Among these, 23 cases (27.71%, 23/83) presented with bilateral lesions. The lesions were unilateral in 58 cases (69.88%, 58/83), including 41 cases (49.40%, 41/83) on the left side and 17 cases (20.48%, 17/83) on the right side. Pleural effusion was observed in 13 cases. All patients recovered after receiving antibiotics, with the exception of one fatality.

### 3.4 Death case

A total of one death was reported, which occurred in November 2022. The deceased was an 81-year-old housewife with a 20-year history of hypertension. On October 11, she purchased a parrot and began caring for it at home, feeding and playing with it daily. On October 22, the patient developed symptoms such as a cough and sore throat but did not seek medical treatment at that time. She was admitted to the hospital for treatment on October 29 after her symptoms worsened on October 27. A chest CT scan revealed an infection in the right lung and a small amount of fluid in both pleural cavities. Sputum samples indicated a *C. psittaci* infection through mNGS testing. On November 5, she died due to psittacosis, which was accompanied by organ failure.

### 3.5 Risk factors associated with *C. psittaci* infection

The study included a total of 319 participants, comprising 80 cases and 239 matched controls. The ratio of cases to controls was ~1:3. All cases exhibited positive results on the mNGS test. No significant demographic differences (such as sex, age, and occupation) were observed between the cases and controls, as shown in Table 2.

**Table 2:** Demographic characteristics of psittacosis cases and controls by sex, age, and occupation in this study.

**Demographic characteristics**	**Cases (*n =* 80), no. (%)**	**Controls (*n =* 139), no. (%)**	**χ^2^**	***P*-value**
Sex			1.02	0.31
Male	40(50.0)	135(56.5)		
Female	40(50.0)	104(43.5)		
Age group (years)			5.56	0.35
< 30	4(5.0)	12(5.0)		
30–39	6(7.5)	25(10.5)		
40–49	9(11.3)	29(12.1)		
50–59	14(17.5)	20(8.4)		
60–69	24(30.0)	76(31.8)		
≥70	23(28.8)	77(32.2)		
Occupation			3.10	0.08
Poultry worker	38(47.5)	87(36.4)		
Non-poultry worker	42(52.5)	152(63.6)		

As shown in the univariable analysis, potential risk factors for infection included the presence of bird habitats within 500 m of the living area, households with backyard birds such as parrots or pigeons, and households with backyard poultry such as chickens or ducks. Other potential risk factors, including poultry consumption, contact with poultry in trade markets, contact with poultry during slaughter, raising pigeons, and interaction with parrots in live bird markets, were not significantly more prevalent among cases than among controls.

Multivariable logistic regression analysis indicated that the presence of bird habitats within 500 m of the living area(*P* < 0.05, OR = 3.81, 95%CI = 2.19–6.61), households with backyard birds such as parrots or pigeons (*P* < 0.05, OR = 2.95, 95%CI = 1.10–7.89) and households with backyard poultry such as chickens or ducks (*P* < 0.05, OR = 2.15, 95% CI = 1.02–4.53) remained significantly associated with risk of disease, as shown in Table 3.

**Table 3:** Potential risk factors associated with *Chlamydia psittaci* infection.

**Variable**	**Cases *n =* 80 (%)**	**Controls *n =* 239 (%)**	**Univariable analysis**		**Multivariable logistic regression analysis**	
			**OR (95% CI)**	***P*** **-value**	**OR (95% CI)**	***P*** **-value**
Presence of bird habitats within 500 m of the living area^a^	40(50.00)	50(20.92)	3.78(2.21–6.47)	0.00	3.81(2.19–6.61)	0.00
Live poultry consumption	17(21.25)	72(30.13)	0.63(0.34–1.14)	0.13		
Live poultry contact in traded markets	5(5.25)	31(12.97)	0.45(0.17–0.19)	0.10		
Households with backyard poultry such as chickens or ducks	16(20.00)	21(8.79)	6.92(3.19–15.02)	0.00	2.15(1.02–4.53)	0.04
Poultry slaughtering	9(11.25)	36(15.06)	0.72(0.33–1.56)	0.40		
Direct contact with sick/dead poultry	4(5.00)	16(6.69)	0.73(0.24–2.26)	0.59		
Parrots contact in live bird market	3(3.75)	4(1.67)	2.29(0.50–10.45)	0.27		
Households with backyard birds such as parrots or pigeons	9(11.25)	10(4.18)	2.90(1.14–7.42)	0.02	2.95(1.10–7.89)	0.03

OR, odds ratios; CI, confidence intervals.

^*a*^Presence of bird habitats within 500 m of the living area: the presence of bird habitats within a 500 m living radius within 2 weeks before the onset of *C. psittaci* infection.

## 4 Discussion

The range of clinical symptoms associated with human psittacosis is extensive and varies significantly, encompassing asymptomatic cases or mild influenza-like illnesses to severe instances of atypical pneumonia, which can occasionally result in fatalities (13). The clinical manifestations of psittacosis resemble those associated with other pathogens (e.g., SARS-CoV-2, seasonal influenza, etc.) (14). The primary symptoms include fever, cough, dyspnea, and chest tightness, with most cases exhibiting unilateral or bilateral pneumonia, similar to findings in other studies (15). In our research, we also observed that the initial symptoms primarily included fever, malaise, and other non-respiratory symptoms, indicating that respiratory symptoms were not the predominant initial presentations. One fatal case had a history of hypertension and diabetes. The cause of death was multi-organ failure attributed to advanced age, highlighting the severe complications associated with psittacosis.

In recent years, the number of reported cases of human psittacosis has increased significantly worldwide (16). This study indicates that the incidence of human psittacosis cases in Hangzhou has risen annually since 2021, with the majority being laboratory-confirmed cases. Furthermore, the median age of individuals affected by psittacosis was 63 years. A substantial proportion of cases involved homemakers, accounting for 46.72% of the total. Additionally, one death was recorded in 2022. A family outbreak was also reported, with two family members infected after exposure to parrots. Austria, Denmark, Germany, Sweden, and the Netherlands have observed a rise in psittacosis cases since November-December 2023, continuing into early 2024 (17). Sweden has suggested that the overall increase in human psittacosis cases may be attributed to the increased use of more sensitive polymerase chain reaction (PCR) panels (18).

There was no significant difference in sex distribution, which differed from the Japanese and Australian studies (19). Although the total number of cases is roughly the same for men and women overall, the distribution was age dependent. Specifically, there were more female cases among individuals aged < 60 years, while male cases predominated among those aged ≥60 years. Similar trends were observed in Japan's national surveillance data, which reported 115 cases of psittacosis from 2007 to 2016, indicating a higher number of female cases among individuals aged < 50 years (20). In contrast, studies conducted in England and Italy revealed an overall male predominance, with female cases being older (21). While these differences between countries may reflect varying exposures to birds, it is important to note that these studies were conducted in the 1980s using complement fixation (CF) tests, and comparisons should be made with caution.

Our current community-based case-control study identified three risk factors for human psittacosis infection: the presence of bird habitats within 500 m of the living area, and the raising of poultry, including chickens, ducks, and parrots. The study also indicates that exposure to parrots and backyard poultry, such as chickens and ducks, were the primary source of psittacosis infection. Previous research has shown that human psittacosis cases are associated with lovebirds and pet birds, such as parrots and cockatoos, which harbor *C. psittaci*. The disease can be contracted by buyers, sales center personnel, and workers at hatcheries (22). Poultry-associated cases have been reported on a poultry farm in France and in rural areas of China, linked to infected chickens, ducks, and geese (23). Direct contact with poultry, particularly during slaughter and processing, increases the risk of infection. Several outbreaks of severe community-acquired pneumonia of *C. psittaci* have also been reported in various countries (24). In this study, the occupations of homemaker and farmer accounted for the majority of cases. Individuals in both occupational categories were more likely to be exposed to and interact with parrots and poultry at home. Some studies have found that social isolation and restrictions on recreational activities due to COVID-19 since 2020 may have led to an increased demand for pets, especially parrots (25). People spent a lot of time at home with their pets, which can elevate the risk of psittacosis. In addition, some studies suggest that human-to-human transmission of *C. psittaci* is an emerging public health concern, particularly among healthcare workers and their close contacts (26). Furthermore, 14 patients in this study denied any history of close contact with live birds or poultry, suggesting that they may have been infected by inhaling *C. psittaci* organisms present in the environment. This environment may have been contaminated by the feces of live birds or poultry infected with *C. psittaci*. But human-to-human transmission cannot be completely ruled out.

It is essential to strengthen surveillance systems to facilitate early detection and rapid response. Although psittacosis is not classified as a notifiable infectious disease in China, some countries require reporting to authorities within 48 h (27). Since 2021, human psittacosis has been designated as a notifiable disease in Hangzhou City, with cases reported to the China Information System for Disease Control and Prevention Infectious Disease Surveillance System. Epidemiological investigations have been conducted to identify potential exposures and outbreaks. Additionally, external environmental surveillance systems have been established, which include laboratory examinations of poultry specimens submitted for avian influenza testing. This analysis aims to monitor the prevalence of *C. psittaci* in poultry on a monthly basis and to provide early warnings of human psittacosis.

Several measures should be proposed to prevent and control psittacosis and to mitigate the effects of the increasing number of patients affected by this disease. Firstly, we need to encourage clinicians to test suspected psittacosis cases for diagnosis using RT-PCR, mNGS or specific antibodies. However, the cost of mNGS is relatively high in clinic, so it cannot completely replace current conventional identification methods, especially in outbreaks. Secondly, we should conduct continuous surveillance of *C. psittaci* in the environments of poultry and wild birds. Thirdly, we should advise bird owners to keep cages clean, ensure that cages are positioned to prevent the transfer of feces between them, and avoid overcrowding (28). Fourthly, owners of caged or domesticated birds and poultry should maintain good personal hygiene and ensure proper ventilation when handling birds, bird droppings, and the surrounding environment. They should take precautions by wearing masks and always wash their hands after handling. Fifthly, ensure that purchase channels are legitimate and avoid bringing home birds from unknown sources. Avoid direct contact with sick birds or poultry.

However, our study has several limitations. First, we only included confirmed cases of human psittacosis because, due to the high cost of testing, physicians only performed mNGS testing on those cases with relatively severe symptoms. In addition, the controls refused to provide throat swabs or blood samples, making it difficult to exclude the possibility of subclinical infection among them. This introduced a selective bias as some cases with atypical symptoms were undetected. Second, our study was unable to establish a dose-response relationship between the frequency of exposure to birds and poultry. Third, there were many older adult participants in our study who could not accurately recall the exact information, such as the date of onset, clinical manifestations, exposure to birds or poultry, which contributed to recall bias.

## 5 Conclusions

In conclusion, we have observed an increasing number of reported human psittacosis cases in Hangzhou City in recent years. Our study shows that *C. psittaci* can be found in poultry such as chickens, ducks and parrots. The transmission of the pathogen from poultry and birds to humans is common and human-to-human transmission is rare. Urgent actions to contain psittacosis cases and mitigate the impact of outbreaks. These actions should include strengthening surveillance, raising public awareness and promoting collaboration between the agricultural and health sectors. In addition, health education on personal hygiene should be provided to the public, especially those who keep poultry and birds.

## Data Availability

The original contributions presented in the study are included in the article/supplementary material, further inquiries can be directed to the corresponding author.
